# The Continued Threat of Influenza A Viruses

**DOI:** 10.3390/v14050883

**Published:** 2022-04-24

**Authors:** Norbert J. Roberts, Leonard R. Krilov

**Affiliations:** 1 Division of Infectious Diseases and Immunology, Department of Medicine, New York University Grossman School of Medicine, New York, NY 10016, USA; 2 Division of Infectious Diseases, Department of Internal Medicine, University of Texas Medical Branch at Galveston, Galveston, TX 77555, USA; 3 Division of Infectious Diseases, Department of Pediatrics, New York University Long Island School of Medicine, Mineola, NY 11501, USA; leonard.krilov@nyulangone.org

Influenza A virus (IAV) is a major cause of respiratory infections worldwide, with the most severe cases occurring in the very young and in elderly individuals [1,2]. Infants and children having their first encounter with the virus have the greatest incidence of severe enough infection to require medical attention. Although the highest illness rates occur in children, most deaths occur with infections of the elderly [2,3]. In addition, immunocompromised individuals of any age are at greater risk of adverse outcomes [4]. The relationship between patient age and mortality differs between seasonal and pandemic IAV infections. Unlike the seasonal IAV age risks noted above, pandemic IAV infections often disproportionately kill young adults, as has been well documented for the 1918 [1,5], 1968 [6], and even the 2009 [7,8,9] pandemics.

Seasonal IAV was responsible for an average of 12,000–52,000 deaths annually between 2010 and 2020 in the U.S. before the onset of the COVID-19 pandemic, according to the Centers for Disease Control and Prevention, with preventive measures addressing the latter respiratory infection also reducing the impact of seasonal influenza. IAV pandemics emerge at unpredictable intervals. There have been four influenza pandemics in the last 103 years, in 1918, 1957, 1968 and 2009. The 1918 H1N1 purely avian virus pandemic was responsible for an estimated 675,000 U.S. deaths and 50 to 100 million deaths worldwide [10,11]. The 1957, 1968 and 2009 pandemic strains were reassortant viruses [12]. Viral strains with pandemic potential continue to be isolated from avian species. For example, outbreaks of human infection by avian influenza A virus subtypes H5N1 and H7N9 have been associated with 53% and 39% mortality, respectively, according to World Health Organization data. Examples of avian IAV that have been transmitted to humans include not only the H5N1 and H7N9 viruses but also H7N7, H9N2, H7N3 and H10N7 viruses [13].

Direct avian to human infections with subsequent person-to-person transmission are possible in view of demonstrated avian mutation risks. Hemagglutinins from circulating clades of avian H5N1 virus require as little as a single base pair mutation to quantitatively switch their binding to human receptors [14]. Of note, avian H5N1 viruses have been isolated in wild birds in the United States and in commercial and backyard poultry in at least 29 states since late 2021 and 2022 to date, marking the first detection of this strain in the US since 2015 (https://www.usgs.gov/centers/nwhc/science/distribution-highly-pathogenic-avian-influenza-north-america-20212022#overview (accessed on 22 April 2022)). No human cases have been reported to date in the U.S. (https://www.cdc.gov/flu/avianflu/spotlights/2021-2022/h5n1-low-risk-public.htm (accessed on 22 April 2022)). The H7N9 hemagglutinin shows limited binding to human receptors; however, if a single amino acid mutation occurs, this would result in structural changes within the receptor binding site that allow extensive binding to human receptors present in the upper respiratory tract [15].

Seasonal IAV epidemics are commonly caused by viruses that arise in and spread from Asia, as documented for H3N2 viruses [16]. Strains with pandemic potential also commonly arise first in Asia, such as the highly pathogenic H5N1 and H7N9 avian viruses mentioned above. IAV can still surprise, however, as illustrated by the emergence of the avian–swine–human reassortant [17] 2009 H1N1 pandemic virus in Mexico, followed by its rapid dissemination worldwide via air travel [18].

Currently, proposed routes for introduction of a new IAV from the avian reservoir into the human population are: (a) direct infection; (b) passage in an intermediate host without reassortment (adaptation); and (c) reassortment in an intermediate host such as pigs or humans [19]. The existence in Asia of a “genetic-reassortment laboratory”—a mix of an unprecedented number of people, pigs, and poultry—was cited in a 2005 article by Osterholm [20]. He reported that, at the time of the 1968 Hong Kong H3N2 pandemic, there were estimated to be 790 million people, 5.2 million pigs, and 12.3 million poultry in China. In 2005, there were estimated to be 1.3 billion people, 508 million pigs, and 13 billion poultry in the country. Osterholm also predicted the likely impact of a 1918-style IAV pandemic [21]. He predicted that if one translated the rate of death associated with the 1918 IAV to that in the current population in 2005, there could be 1.7 million deaths in the United States and 180 million to 360 million deaths globally. The subsequent IAV pandemic, in 2009, fortunately did not exhibit that degree of lethality. The risk of a highly lethal IAV pandemic nevertheless continues.

Potential pandemic strains of IAV can also arise as a threat through human error in laboratory quality assurance testing. In March 2005, an IAV H2N2 virus was identified by a local laboratory in Canada when found in lab test kits for quality control. The kits had been distributed to several thousand laboratories in 18 countries. The H2N2 virus that was identified was found to be similar to H2N2 viruses that circulated in humans in 1957–1958 at the beginning of the so-called “Asian” influenza pandemic. That H2N2 virus was fully transmissible among humans and caused almost 70,000 deaths in the U.S. and more than a million deaths worldwide in 1957. It continued to circulate in humans and cause annual epidemics until 1968, when it disappeared after the emergence of the “Hong Kong” IAV H3N2 virus that caused the next pandemic [22,23]. As a result, persons born after 1968 are expected to have little or no immunity to an H2N2 virus. This has been demonstrated by testing sera from Rochester NY and from Hong Kong, measuring antibodies to H2N2 IAV, from individuals born before 1957, or between 1957 and 1968, or after 1968. Sera from those born after 1968 showed no evidence of immunity to an H2N2 virus [24]. More than two-thirds of the world population, including 67.9% of the U.S. population in 2016, were born after 1968, and therefore never exposed to a readily person-to-person transmissible H2N2 virus [25]. The H2N2 viruses were rapidly destroyed by all of the recipient laboratories and a recurrent H2N2 pandemic was averted.

Avian flyways for the wild waterfowl that serve as the natural reservoir for IAV are now supplemented by extensive global air traffic [26], with the ability to spread a viral agent faster than its incubation period. The impact of the waterfowl avian flyways, even in this era of extensive global airline traffic, should not be underestimated, as illustrated by the delivery of highly pathogenic Eurasian avian H5N1 virus to commercial and backyard avian flocks in multiple states currently, in 2022, resulting in culling of flocks and, of course, potential risk of transmission to humans, as noted above. The 1918 highly pathogenic avian virus-related pandemic is usually cited as being first documented in Kansas, and it is not unreasonable to speculate that it was transported to the area via known wild waterfowl flyways.

The impact of human air travel on dissemination of IAV can be demonstrated both for pandemic [18] and non-pandemic [27] strains. For example, in an inter-pandemic period, the effect of travel on IAV epidemiology has been studied by Belderok et al. [28]. They noted that in temperate climates, IAV infection is seasonal, but in the tropics, the destination of many short-term travelers, IAV circulates at low levels year-round, as also documented by others [28,29]. They studied 1190 adult short-term travelers from the Netherlands to tropical and subtropical countries prospectively. The attack rate for all infections was 7% and for influenza-like illness (ILI), 0.8%. In 15 travelers with fever or ILI, IAV infection was serologically confirmed; 7 of these travelers were considered contagious or to be incubating the infection while traveling home. Given the large number of travelers to tropical countries, they concluded that travel-related infection most likely contributes to importation and further IAV spread worldwide.

Two independent studies from London and Atlanta [30,31] indicated that an IAV pandemic could possibly be contained at its source if there was quick detection, spread was not too fast, sufficient antivirals were deployed quickly and massively around the epicenter, and strict quarantine and other measures were employed. The containment strategy would have to be in place within a few weeks of infection with a virus that was capable of sustained human-to-human transmission. The studies noted that if such a virus were to arise today, that would be unlikely to happen. As noted by Osterholm in 2005, if an IAV pandemic struck today, borders would close, the global economy would shut down, international vaccine supplies and health-care systems would be overwhelmed, and panic would reign. He stated that the reality of a coming pandemic cannot be avoided; only its impact can be lessened [21]. The current COVID-19 pandemic, caused by SARS-CoV-2, very well illustrates the points made by such studies and reports. Furthermore, Dr. Michael Ryan, WHO Executive Director of Health Emergencies, at a WHO Daily press conference on the novel SARS-CoV-2 coronavirus on 28 December 2020, indicated that although the coronavirus pandemic has been “very severe…it is not necessarily the big one…its current case fatality is reasonably low in comparison to other emerging diseases.” The impact of a potential IAV H5N1 pandemic would likely greatly exceed even the U.S. and worldwide devastation due to COVID-19.

IAV remains a continuing threat, and the aim of this Special Issue of *Viruses* regarding “Influenza A Viruses; New Insights in 2022” is to contribute to the current knowledge regarding innate and adaptive immunity to IAV, the pathogenesis of the infection, the ecology of the infection, the populations at risk and epidemiology of the infection, treatment approaches directed at IAV or modification of host responses, and approaches to vaccine development that are likely to benefit the host upon subsequent natural challenge. Approaches to develop broad protective immunity, to current circulating strains and potential pandemic strains, are especially encouraged.

## Data Availability

Not applicable.
